# Prevalence of Sexual Violence and Intimate Partner Violence Among US Military Veterans: Findings from Surveys with Two National Samples

**DOI:** 10.1007/s11606-023-08486-9

**Published:** 2023-11-27

**Authors:** Katherine M. Iverson, Whitney S. Livingston, Dawne Vogt, Brian N. Smith, Shannon M. Kehle-Forbes, Karen S. Mitchell

**Affiliations:** 1 Women’s Health Sciences Division of the National Center for PTSD, VA Boston Healthcare System, Boston, MA USA; 2 Department of Psychiatry, Boston University Chobanian & Avedisian School of Medicine, Boston, MA USA; 3 Center for Care Delivery & Outcomes Research, Minneapolis VA Healthcare System, Minneapolis, MN USA; 4 Department of Medicine, University of Minnesota, Minneapolis, MN USA

**Keywords:** abuse, assault, gender, military, sexual orientation

## Abstract

**Background:**

Sexual violence (SV) and intimate partner violence (IPV) experiences are major social determinants of adverse health. There is limited prevalence data on these experiences for veterans, particularly across sociodemographic groups.

**Objective:**

To estimate the prevalence of SV before, during, and after military service and lifetime and past-year IPV for women and men, and explore differences across sociodemographic groups.

**Design:**

Data are from two national cross-sectional surveys conducted in 2020. Weighted prevalence estimates of SV and IPV experiences were computed, and weighted logistic regression models were used for comparisons across gender, race, ethnicity, sexual orientation, and age.

**Participants:**

Study 1 included veterans of all service eras (*N* = 1187; 50.0% women; 29% response rate). Study 2 included recently separated post-9/11 veterans (*N* = 1494; 55.2% women; 19.4% response rate).

**Main Measures:**

SV was assessed with the Deployment Risk and Resilience Inventory-2 (DRRI-2). IPV was assessed with the extended Hurt-Insult-Threaten-Scream Tool.

**Key Results:**

Women were more likely than men to experience pre-military SV (study 1: 39.9% vs. 8.7%, OR = 6.96, CIs: 4.71–10.28; study 2: 36.2% vs. 8.6%, OR = 6.04, CIs: 4.18–8.71), sexual harassment and/or assault during military service (study 1: 55.0% vs. 16.8%, OR = 6.30, CIs: 4.57–8.58; study 2: 52.9% vs. 26.9%, OR = 3.08, CIs: 2.38–3.98), and post-military SV (study 1: 12.4% vs. 0.9%, OR = 15.49, CIs: 6.42–36.97; study 2: 7.5% vs. 1.5%, OR = 5.20, CIs: 2.26–11.99). Women were more likely than men to experience lifetime IPV (study 1: 45.7% vs. 37.1%, OR = 1.38, CIs: 1.04–1.82; study 2: 45.4% and 34.8%, OR = 1.60, CIs: 1.25–2.04) but not past-year IPV (study 1: 27.9% vs. 28.3%, OR = 0.95, CIs: 0.70–1.28; study 2: 33.1% vs. 28.5%, OR = 1.24, CIs: 0.95–1.61). When controlling for gender, there were few differences across other sociodemographic groups, with the exception of sexual orientation.

**Conclusions:**

Understanding veterans’ experiences of SV and IPV can inform identification and intervention efforts, especially for women and sexual minorities.

Sexual (SV) and intimate partner violence (IPV) are endemic population health problems that profoundly impact the physical and mental health and functioning of the US population,^1^ particularly among vulnerable subgroups such as military veterans.^2–5^ SV includes sexual activity in which consent is not obtained or freely given (i.e., rape, being made to touch someone, sexual coercion, and/or unwanted sexual contact).^6^ SV that occurs during military service, including sexual assault and/or harassment, is termed military sexual trauma (MST).^7^ IPV includes physical, psychological, and/or sexual violence from past or current intimate partners.^8^ Prior research indicates that men and women veterans experience higher rates of SV in childhood than non-veterans^9, 10^ and women veterans are more likely to experience SV and IPV during their lifetime than non-veteran women.^11, 12^

The scope of veterans’ experiences of SV and IPV is not well understood. Most studies have focused on patient and/or clinic-based, rather than population-based, samples (e.g., Veterans Health Administration [VHA] patients).^13–17^ Additionally, little research has examined both lifetime and recent IPV experiences along with SV before, during, and after military service. While some studies have examined SV during these three timeframes, they did not include men or examine sociodemographic factors.^3, 14, 18^ Moreover, MST history has been examined among sexual minorities^19^ and across different age groups,^20^ but these studies were limited to women or only one type of violence.

Research suggests that lifetime IPV prevalence is higher in women than men veterans (i.e., 58% vs. 12.6%).^21^ However, these estimates are primarily derived from convenience and clinical samples which can inflate prevalence rates, and data are very limited for men. Past-year IPV estimates from regional and national surveys also vary widely (e.g., 13–33% for women and 15–37% for men veterans),^22–24^ leaving unresolved the question of what the prevalence estimates are nationally for women and men.

Post-9/11 veterans are those who have served after September 2001 and are an important subgroup for understanding IPV, as some evidence suggests the prevalence may be particularly high in this group.^25, 26^ However, this may reflect the younger age of this cohort, as younger age has been associated with higher risk for IPV.^16, 27, 28^ Veterans’ risk for IPV by other sociodemographic groups such as race, ethnicity, and sexual orientation is less clear because of a lack of research. Although one study found racial minoritized women had higher odds of past-year IPV,^15^ two other studies found that neither race nor ethnicity increased past-year IPV risk among women veterans.^16, 28^ A national survey of women VHA patients found higher prevalence of past-year IPV among sexual minority women (24.7%) compared to heterosexual women (18.0%),^16^ consistent with findings from a regional survey demonstrating higher rates of past-year and lifetime IPV.^29^ Finally, most research examining IPV among veterans by sociodemographic characteristics (e.g., age and race) have focused on women.^21^

To address these gaps, in this retrospective study, we analyzed data collected in 2020 by study authors from two national surveys of US veterans^30, 31^ to accomplish two primary aims: (1) identify prevalence of SV experience before, during, and after military service by gender, and (2) identify prevalence of lifetime and past-year IPV experience by gender. We also explored the likelihood of SV and IPV by other sociodemographic characteristics (i.e., sexual orientation, race, ethnicity, and age). We hypothesized that women would have the highest proportions of SV and IPV compared to men.

## METHODS

### Participants

#### Study 1 (Veterans of all Eras)

A national sample of US veterans of all service eras was recruited to participate in a primarily online cross-sectional survey study examining eating behaviors, military experiences, and healthcare needs.^31^ Potential participants were randomly selected from the US population of veterans by the Veterans Affairs/Department of Defense Identity Repository (VADIR). Women were oversampled for a 1:1 ratio of women to men. Of 4126 veterans identified, 4072 had locatable mailing addresses. We received completed surveys from 1187 participants (29% response rate).

Of the 1187 survey responders, 541 identified as male, 594 identified as female, six identified as other gender categories, and 46 participants left this question blank. The mean age of participants was 53.86 (*SD* = 13.79, range: 19–92). The majority of participants were White (71.2%), with smaller proportions identifying as Black (13.2%), American Indian or Alaska Native (1.3%), Asian (1.3%), Native Hawaiian or Pacific Islander (0.4%), multiple races (4.5%), or other race (3.5%); 7.8% were Hispanic/Latinx.

#### Study 2 (Post-9/11 Veterans)

Study 2 recruited a national sample of post-9/11 US veterans to participate in an online cross-sectional survey examining veterans’ military service and healthcare needs^30^ using the same survey methodology as study 1. VADIR was used to randomly select potential participants from the population of veterans who had separated from service within the prior 18 months. Women were oversampled for a 1:1 ratio of women to men. Of 7700 veterans identified, 7687 had locatable mailing addresses. Surveys were completed by 1494 participants (19.4% response rate).

Of the 1494 survey responders, 565 identified as male, 825 were female, 10 identified as other gender categories, and 94 participants left this question blank. Participants’ mean age was 29.25 (*SD* = 8.23, range: 18–71). The majority of participants were White (69.6%), with smaller proportions identifying as Black (16.9%), American Indian or Alaskan Native (3.8%), Asian (4.8%), Pacific Islander or Native Hawaiian (0.7%), or other (4.4%); 16.1% were Hispanic/Latinx.

#### Procedure (Study 1 and Study 2)

Westat research firm mailed invitations to potential participants, including an informed consent factsheet, an opt-out mechanism, URL and unique code for accessing the online survey, and a $2 bill to keep regardless of participation. Respondents received an additional $20. Potential participants received up to three reminder postcards. Paper surveys were sent to those who did not respond/opt-out; the final reminder was sent after mailing the paper survey. Surveys were administered between February and May 2020. Additional methodological details are reported elsewhere.^30, 31^ Reports of violence did not differ significantly in study 2 for participants who completed the survey prior to COVID-19-related lockdowns (before March 15, 2020) or after lockdowns began (March 15, 2020, or later). The only significant difference in the study 1 sample was that 15.9% of participants who completed the survey before the lockdowns reported pre-military SV, compared to 8.8% of participants who completed the survey after lockdowns began. Study procedures were approved by the VA Boston Healthcare System’s Institutional Review Board.

#### Measures (Study 1 and Study 2)

*Pre-military SV* was assessed using the Prior Stressors scale of the Deployment Risk and Resilience Inventory-2 (DRRI-2).^32^ A positive response to either the item asking about SV during childhood or the item asking about SV during adulthood indicated pre-military SV experience (yes/no). *SV during military service* (*i.e*., *MST*) was assessed using the DRRI-2’s Sexual Harassment scale.^32^ Instructions were modified to focus on the entire military service period. We examined MST (sexual assault and/or sexual harassment; yes/no) and military sexual assault (yes/no). *Post-military SV* was assessed using the DRRI-2’s Postdeployment Stressors scale.^32^ A positive response to an item asking about SV since separation from service indicated post-military SV (yes/no).

*Lifetime and past-year IPV* were assessed using the extended HITS (*Hurt-Insult-Threaten-Scream*) *Tool*.^33, 34^ The original HITS consisted of 4 items; the extended version includes an additional item assessing sexual IPV using VHA’s sexual coercion screening item. The following items were asked for past year and prior to the past year: “How often did your partner:^1^ physically hurt you?,^2^ insult you or talk down to you?,^3^ threaten you with harm?,^4^ scream or curse at you?, and^5^ force or pressure you to have sexual contact against your will, or when you were unable to say no.” Responses are on a 5-point scale (1 = never to 5 = frequently). Items were summed for a total score. Scores ≥ 7 indicated IPV (yes/no).^35^

##### Sociodemographic Variables

Participants self-reported their gender (male, female, transgender man, transgender woman, genderqueer, other gender category, something else), sexual orientation (heterosexual/straight, gay/lesbian, bisexual, something else), race, ethnicity, and age. For sexual orientation, we distinguished between gay/lesbian veterans and bisexual veterans because bisexual individuals may experience more traumas, including lifetime IPV, than gay/lesbian individuals^36, 37^ and SV research in military populations calls for a distinction between gay/lesbian and bisexual individuals.^38^

### Statistical Analyses (Study 1 and Study 2)

Prevalence estimates were calculated using the “survey” package in R 4.0.0. We used a weighting class method^39^ to calculate sample weights to obtain more nationally representative prevalence estimates and more precise standard errors for each study sample to enhance representativeness to the general veteran population for study 1^31^ and the post-9/11 veterans population for study 2.^30^ All weighting steps were done separately by gender. The first step calculated a base weight for each sampled case, which is the reciprocal of the case’s selection probability. The second step adjusted the base weights for nonresponse by using data available for every record in VADIR, including sex, service component, personnel category, pay grade, race, ethnicity, marital status, education level, region, state, presence of address on file, and presence of address on Lexus Nexus database. The adjusted base weights for respondents are base weights multiplied by the corresponding adjustment factor, whereas the adjusted base weight for nonrespondents is zero. The final step computes adjustments to ensure the weighted respondents correspond to expected proportions from the population. Weights were developed by sex within each study population; concatenating male and female files together properly represents the proportions of males and females in the population. The final weight is appropriate for analyzing by sex or across sex.

Weighted logistic regression models were used to compare the odds of SV and IPV across sociodemographic groups by gender, race, ethnicity, sexual orientation, and age. Models were estimated separately for each sociodemographic subgroup. Analyses comparing violence exposures across genders were conducted among only participants identifying as men or women, given the small numbers of participants identifying as other genders. For our primary aims focused on gender (aims 1 and 2), we controlled the false discovery rate (FDR) using the Benjamini–Hochberg procedure.^40^ We set the FDR equal to 0.05 and calculated adjusted *p*-values for this family of tests for both samples. For our exploratory analyses, in models with sexual orientation, race, or ethnicity as the independent variable, we adjusted these models for gender (man or woman). Analyses comparing violence exposures across racial groups were conducted among participants who identified as White, non-Hispanic/Latinx only, Black, non-Hispanic/Latinx only, and Hispanic/Latinx. Gender-stratified models were used to estimate the impact of age group (18–44 or ≥ 45) on violence exposures.

## Results

### Study 1

Over half of women experienced MST (harassment and/or assault). Women were more likely to experience SV at each timeframe and lifetime IPV, but not past-year IPV, compared to men (Table 1). After adjusting for gender, gay/lesbian participants and bisexual participants were more likely to experience pre-military SV than heterosexual participants and bisexual participants were more likely to report lifetime IPV (Table 2). The prevalence of SV and IPV was similar across racial and ethnic groups as well. Black participants were more likely to report past-year IPV compared to White participants (Table 3). There were no differences in odds of experiencing SV and IPV across age groups among women. Younger men were more likely to experience lifetime and past-year IPV compared to older men (Table 4).

**Table 1:** Prevalence of sexual violence and IPV experiences by gender in a national sample of veterans

	Full Weighted %	Women (*n* = 594) Weighted %	Men (*n* = 541) Weighted %	Logistic regression models comparing men and women		
				*p*	Adjusted *p*	OR (95% CI)
Pre-military sexual violence	12.5	39.9	8.7	< 0.001	0.001	6.96 (4.71,10.28)
MSA	6.1	25.6	3.1	< 0.001	0.001	11.02 (5.53, 22.20)
MST	21.8	55.0	16.8	< 0.001	0.001	6.30 (4.57, 8.58)
Post-military sexual violence	2.4	12.4	0.9	< 0.001	0.001	15.49 (6.42, 36.97)
Lifetime IPV	36.7	45.7	37.1	0.03	0.04	1.38 (1.04, 1.82)
Past-year IPV	27.2	27.9	28.3	0.76	0.76	0.95 (0.70, 1.28)

*IPV* intimate partner violence, *MSA* military sexual assault, *MST* military sexual trauma (includes sexual assault and/or harassment). Sample weights were applied to estimate prevalence and logistic regression models. *p*-values were adjusted using the Benjamini–Hochberg procedure for controlling the false discovery rate

**Table 2:** Prevalence of sexual violence and IPV experiences by sexual orientation in a national sample of veterans

	Heterosexual (*n* = 1056) Weighted %	Gay or lesbian (*n* = 39) Weighted %	Logistic regression models comparing gay or lesbian veterans to heterosexual veterans		Bisexual (*n* = 30) Weighted %	Logistic regression models comparing bisexual veterans to heterosexual veterans	
			*p*	OR (95% CI)		*p*	OR (95% CI)
Pre-military sexual violence	11.2	54.1	0.01	6.95 (1.54, 31.37)	59.6	< 0.01	12.62 (2.44, 65.28)
MSA	5.7	9.1	0.52	0.76 (0.33, 1.74)	27.0	0.65	0.79 (0.29, 2.14)
MST	20.8	46.4	0.31	2.16 (0.48, 9.67)	55.4	0.47	1.58 (0.46, 5.35)
Post-military sexual assault	2.3	3.9	0.31	0.44 (0.09, 2.14)	9.9	0.45	1.56 (0.50, 4.86)
Lifetime IPV	38.4	25.4	0.21	0.46 (0.14, 1.58)	52.7	0.04	2.78 (1.04, 7.36)
Past-year IPV	28.5	17.2	0.42	0.55 (0.12, 2.40)	33.0	0.24	1.89 (0.66, 5.46)

*IPV* intimate partner violence, *MSA* military sexual assault, *MST* military sexual trauma (includes sexual assault and/or harassment). Sample weights were applied to estimate prevalence and logistic regression models. Models adjusted for gender. The breakdown of participants by sexual orientation and gender is as follows: heterosexual (524 women, 524 men); gay or lesbian (29 women, 7 men); bisexual (26 women, 2 men); something else (5 women, 1 men)

**Table 3:** Prevalence of sexual violence and IPV experiences by race and ethnicity in a national sample of veterans

	White (*n* = 800) Weighted %	Black (*n* = 152) Weighted %	Logistic regression models comparing White and Black veterans		Non-Latinx (*n* = *1043*) Weighted %	Latinx (*n* = 92) Weighted %	Logistic regression models comparing Latinx and non-Latinx veterans	
			*p*	OR (95% CI)			*p*	OR (95% CI)
Pre-military sexual violence	10.8	15.4	0.48	1.34 (0.60, 2.97)	12.8	12.3	0.92	1.05 (0.42, 2.60)
MSA	5.2	10.5	0.22	2.03 (0.65, 6.28)	6.0	6.7	0.76	1.29 (0.25, 6.79)
MST	21.2	23.0	0.93	0.97 (0.49, 1.91)	22.0	17.8	0.63	0.81 (0.35, 1.89)
Post-military sexual violence	2.0	3.7	0.36	1.58 (0.59, 4.20)	2.3	4.1	0.22	2.27 (0.61, 8.49)
Lifetime IPV	37.3	46.6	0.12	1.59 (0.89, 2.86)	38.3	36.0	0.74	0.88 (0.43, 1.83)
Past-year IPV	24.8	41.8	0.01	2.35 (1.29, 4.28)	27.6	32.4	0.60	1.23 (0.57, 2.62)

*IPV* intimate partner violence, *MSA* military sexual assault, *MST* military sexual trauma (includes sexual assault and/or harassment). Sample weights were applied to estimate prevalence and logistic regression models. Models adjusted for gender

**Table 4:** Prevalence of sexual violence and IPV by age in a national sample of veterans

	Women					Men			
		Aged 18–44 (*n* = 201) Weighted %	Aged ≥ 45 (*n* = 389) Weighted %	Logistic regression models comparing women aged 18–44 and women aged ≥ 45		Aged 18–44 (*n* = 104) Weighted %	Aged ≥ 45 (*n* = 433) Weighted %	Logistic regression models comparing men aged 18–44 and men aged ≥ 45	
				*p*	OR (95% CI)			*p*	OR (95% CI)
Pre-military sexual violence		37.7	42.1	0.32	1.21 (0.83, 1.76)	13.5	7.2	0.07	0.50 (0.24, 1.06)
MSA		25.3	26.2	0.69	1.09 (0.72, 1.66)	3.9	1.9	0.31	0.48 (0.12, 2.00)
MST		54.3	56.3	0.42	1.17 (0.81, 1.69)	20.6	14.6	0.15	0.64 (0.35, 1.17)
Post-military sexual violence		8.9	14.8	0.07	1.76 (0.96, 3.24)	0.7	1.0	0.75	1.43 (0.16, 12.66)
Lifetime IPV		48.0	44.4	0.61	0.91 (0.63, 1.31)	47.7	33.3	0.03	0.56 (0.33, 0.95)
Past-year IPV		32.3	25.0	0.09	0.71 (0.48, 1.06)	39.5	24.1	0.01	0.48 (0.28, 0.83)

*IPV* intimate partner violence, *MSA* military sexual assault, *MST* military sexual trauma (includes sexual assault and/or sexual harassment). Sample weights were applied to estimate prevalence and logistic regression models

### Study 2

As with Study 1, over half of women experienced MST, and women were more likely to experience SV at each timeframe as well as lifetime IPV, but not past-year IPV, compared to men (Table 5). After adjusting for gender, there were no differences in odds of experiencing exposures between veterans identifying as gay/lesbian compared to heterosexual veterans. However, veterans identifying as bisexual were significantly more likely to experience pre-military SV, military sexual assault, lifetime IPV, and past-year IPV compared to heterosexual veterans (Table 6). The only difference across racial groups was that Black veterans were significantly more likely to experience pre-military SV compared to White veterans; there were no differences by ethnicity (Table 7). There were no differences in the odds of violence exposures by age group in either women or men (Table 8).

**Table 5:** Prevalence of sexual violence and IPV experiences by gender in a national sample of post-9/11 veterans

	Full Weighted %	Women (*n* = 825) Weighted %	Men (*n* = 565) Weighted %	Logistic regression models comparing men and women		
				*p*	Adjusted *p*	OR (95% CI)
Pre-military sexual violence	13.8	36.2	8.6	< 0.001	0.001	6.04 (4.18, 8.71)
MSA	7.4	22.7	4.1	< 0.001	0.001	6.82 (4.04, 11.51)
MST	31.4	52.9	26.9	< 0.001	0.001	3.08 (2.38, 3.98)
Post-military sexual violence	2.6	7.5	1.5	< 0.001	0.001	5.20 (2.26, 11.99)
Lifetime IPV	34.3	45.4	34.8	< 0.001	0.001	1.60 (1.25, 2.04)
Past-year IPV	27.3	33.1	28.5	0.11	0.11	1.24 (0.95, 1.61)

*IPV* intimate partner violence, *MSA* military sexual assault, *MST* military sexual trauma (includes sexual assault and/or sexual harassment). Sample weights were applied to estimate prevalence and logistic regression models. *p*-values were adjusted using the Benjamini–Hochberg procedure for controlling the false discovery rate

**Table 6:** Prevalence of sexual violence and IPV experiences by sexual orientation in a national sample of post-9/11 veterans

	Heterosexual (*n* = 1201) Weighted %	Gay or lesbian (*n* = 65) Weighted %	Logistic regression models comparing gay or lesbian veterans to heterosexual veterans		Bisexual (*n* = 108) Weighted %	Logistic regression models comparing bisexual veterans to heterosexual veterans		
			*p*	OR (95% CI)			*p*	OR (95% CI)
Pre-military sexual violence	11.3	35.0	0.15	2.37 (0.73, 7.63)	53.2		< 0.001	4.11
MSA	6.3	17.2	0.42	1.57 (0.53, 4.64)	32.0		0.04	2.85 (1.05, 7.75)
MST	30.5	50.2	0.21	1.72 (0.74, 4.01)	58.0		0.15	1.71 (0.82, 3.57)
Post-military sexual violence	2.2	7.3	0.32	2.20 (0.46, 10.48)	9.1		0.05	1.94 (0.99, 3.80)
Lifetime IPV	36.0	32.1	0.42	0.74 (0.35, 1.54)	62.2		0.01	2.36 (1.18, 4.69)
Past-year IPV	28.6	25.3	0.59	0.80 (0.36, 1.77)	48.8		0.02	2.27 (1.17, 4.40)

*IPV* intimate partner violence, *MSA* military sexual assault, *MST* military sexual trauma (includes sexual assault and/or sexual harassment). Sample weights were applied to estimate prevalence and logistic regression models. Models adjusted for gender. The breakdown of participants by sexual orientation and gender as follows: heterosexual (656 women, 540 men); gay or lesbian (51 women, 11 men); bisexual (98 women, 7 men); something else (9 women, 4 men)

**Table 7:** Prevalence of sexual violence and IPV experiences by race and ethnicity in a national sample of post-9/11 veterans

	White (*n* = 821) Weighted %	Black (*n* = 185) Weighted %	Logistic regression models comparing White and Black veterans		Non-Latinx (*n* = 1161) Weighted %	Latinx (*n* = 241) Weighted %	Logistic regression models comparing Latinx and non-Latinx veterans	
			*p*	OR (95% CI)			*p*	OR (95% CI)
Pre-military sexual violence	10.3	23.8	0.04	2.06 (1.02, 4.16)	13.0	15.4	0.57	1.16 (0.69, 1.94)
MSA	7.1	8.3	0.59	0.78 (0.31, 1.95)	7.6	5.4	0.08	0.62 (0.37, 1.06)
MST	33.2	27.1	0.07	0.58 (0.32, 1.03)	32.4	27.9	0.21	0.77 (0.51, 1.16)
Post-military sexual violence	2.2	4.3	0.57	1.60 (0.32, 7.87)	2.1	3.6	0.21	1.69 (0.74, 3.86)
Lifetime IPV	39.5	32.0	0.13	0.66 (0.39, 1.12)	37.5	31.9	0.16	0.75 (0.50, 1.12)
Past-year IPV	30.3	26.5	0.44	0.80 (0.46, 1.40)	29.6	26.5	0.43	0.84 (0.55,1.29)

*IPV* intimate partner violence, *MSA* military sexual assault, *MST* military sexual trauma (includes sexual assault and/or sexual harassment). Sample weights were applied to estimate prevalence and logistic regression models. Models adjusted for gender

**Table 8:** Prevalence of sexual violence and IPV experiences by age in a national sample of post-9/11 veterans

	Women				Men			
	Aged 18–44 (*n* = 780) Weighted %	Aged ≥ 45 (*n* = 36) Weighted %	Logistic regression models comparing women aged 18–44 and women aged ≥ 45		Aged 18–44 (*n* = 533) Weighted %	Aged ≥ 45 (*n* = 29) Weighted %	Logistic regression models comparing men aged 18–44 and men aged ≥ 45	
			*p*	OR (95% CI)			*p*	OR (95% CI)
Pre-military sexual violence	35.7	39.3	0.70	1.16 (0.54, 2.49)	8.1	20.3	0.09	2.86 (0.84, 9.75)
MSA	22.4	24.3	0.81	1.11 (0.48, 2.55)	4.3	0.0	**–**	**–**
MST	52.6	57.3	0.65	1.19 (0.56, 2.51)	26.8	35.9	0.41	1.53 (0.56, 4.20)
Post-military sexual violence	7.6	5.7	0.68	0.72 (0.15, 3.44)	1.6	0.0	**–**	**–**
Lifetime IPV	45.5	40.5	0.57	0.81 (0.38, 1.70)	34.7	32.9	0.81	0.88 (0.34, 2.33)
Past-year IPV	33.3	17.5	0.07	0.43(0.17, 1.08)	25.8	29.9	0.94	1.04 (0.38, 2.86)

*IPV* intimate partner violence, *MSA* military sexual assault, *MST* military sexual trauma (includes sexual assault and/or sexual harassment). Sample weights were applied to estimate prevalence and logistic regression models

## DISCUSSION

This study investigated prevalence of SV before, during, and after military service, in addition to lifetime and past-year IPV, by gender among the general veteran population and post-9/11 veterans. This study also examined prevalence estimates of these experiences among racially and ethnically marginalized communities and sexual minoritized veterans, providing insight into the violent experiences that these communities oftentimes face given underrepresentation in previous research. It is difficult to compare the current estimates of SV and IPV to prior studies, as methodological differences across studies contribute to wide variability in estimates.^21, 41^ Yet, results underscore the importance of screening and identification policies to include childhood and adult SV and IPV across the life course, and to consider particular groups at risk for these experiences, especially women and possibly sexual minority veterans.

Prior research consistently demonstrates that women experience a higher burden of SV and lifetime IPV than men.^42, 43^ Extending previous findings regarding SV,^5, 44^ this study demonstrated that women were three (post-9/11 women) to six (women of all eras) times more likely to experience MST than men. The smaller gender difference among the post-9/11 sample likely reflects the relatively high proportion of men reporting MST among this younger group (26.9%) compared to the general veteran sample (16.8%). Gender differences were particularly pronounced for military sexual assault, and women were more likely to experience pre- and post-military SV and lifetime IPV in both samples. This heightened risk for SV and IPV among women is concerning as some studies have found the associations between MST and mental health impacts and suicide risk are stronger among women than men.^4, 45, 46^ Similarly, lifetime IPV is strongly associated with mental health difficulties and doubles the risk of suicidal ideation and attempts among women.^47, 48^

This study extends the literature by identifying estimates of IPV among veteran men, an understudied group with respect to IPV experiences.^21^ Proportions of lifetime and past-year IPV were 37.1% and 28.3% among men veterans from all eras, and 34.8% and 28.5% among post-9/11 men veterans. Women were more likely to experience lifetime IPV in both samples, but men and women did not differ in likelihood of experiencing past-year IPV. These findings are generally consistent with prior research examining recent IPV with samples that were not nationally representative.^28, 29^ It should be noted that prevalence does not account for violence severity, and women experience more severe IPV and more health-related impacts.^1^ Nonetheless, understanding the prevalence of IPV among men is important because they are sometimes not considered at risk of experiencing IPV, given the potential for power differentials and historical messages of male dominance in intimate relationships.^49, 50^ However, men have negative health outcomes following IPV,^25, 27^ and face sociocultural stereotypes about masculinity that increase stigma and shame around disclosure, which may be elevated by military culture.^27, 51^ Given the high prevalence of IPV among veteran men, efforts to reduce stigma and barriers to treatment may increase access to IPV-related care (i.e., PTSD treatment)^52, 53^ while screening and intervention services remain vital for all veterans.^54^

Black veterans were more likely than White veterans to report past-year IPV in the sample of veterans of all eras, while Black veterans in the post-9/11 sample were more likely to experience pre-military SV. Research suggests systematic racialized oppression and socioeconomic factors as explanatory variables for the high rates of IPV and childhood SV among Black people in general.^55–57^ Screening for lifetime SV and past-year IPV experiences may be important among Black veterans as they experience greater somatic rather than cognitive symptoms in response to trauma.^58^ Thus, PTSD screening may not capture these veterans who are at increased risk for premilitary SV and recent IPV. Moreover, providers could take a race-conscious approach to screening patients of racially and ethnically marginalized identities given the racism and harmful stigma often encountered in the medical system.^59, 60^ These differences can be understood as an outcome of structural racism, rather than a reflection of race-specific characteristics. This study extends the literature by exploring SV, MST, and IPV among Black and Hispanic veterans; however, these findings are preliminary and additional research oversampling for veterans of other racial and ethnic marginalized identities is needed.

Findings from both samples indicate that gay or lesbian veterans relative to heterosexual veterans were more likely to experience MST. Even after adjusting for gender, overall patterns of findings across the two samples provide evidence of further risk for SV and IPV among bisexual veterans relative to heterosexual veterans. Heightened exposure to these forms of violence may help explain why these individuals have twice the risk of PTSD, depression, and physical health symptoms than heterosexual veterans.^61^ Research should attempt to replicate these findings with larger samples.

Only one difference in violence was observed across age groups. Younger men in the general sample of veterans were more likely to experience lifetime and past-year IPV relative to their older counterparts. Among post-9/11 veterans, differences were not observed across age groups, which likely reflects a ceiling effect as 94.8% of the men in that sample were below age 45 compared to only 19.4% of the men in the general sample. The lack of differences by age in past-year IPV among women contrasts with prior research.^16, 27, 28^ Additional research on violence exposure by age is needed but findings may reinforce the need to screen for SV and IPV beyond reproductive age.

Limitations of this investigation include the use of dichotomous SV and IPV, which neglect important contextual factors (e.g., severity, chronicity, and impacts). It is unknown to what extent SV was perpetrated by intimate partners, and thus, overlaps with IPV (i.e., SV at any timepoint may have been perpetrated by an intimate partner). Although veterans who identify as gay, lesbian, and bisexual may be at particular risk for SV and IPV, the subsamples were small and uneven and analyses were exploratory; caution is warranted when generalizing findings to sexual minority veterans. Additional research is necessary to understand these prevalence estimates among veterans of other sex and gender marginalized communities. The surveys did not assess SV in past-year or IPV at the pre-/during-/post-military timepoints. Similarly, we focused on several key sociodemographic factors; however, future research should examine variation in violence across additional factors (e.g., gender minority and socioeconomic status, military rank). Response rates may have been impacted by the COVID-19 pandemic, but they were consistent with previous survey studies of veterans.^62, 63^ Further, the use of sample weights to adjust for non-response bias helps attenuate this concern. Nonetheless, non-response rates may introduce bias. As participants were informed the studies were about military experiences and health needs, it is possible that those who had violent experiences during military service were less likely to participate.

Clinicians treating military and veteran populations should be knowledgeable of these prevalence estimates and risk groups and be prepared to provide trauma-informed screening, assessment, and intervention. VHA screens all patients for MST^64^ and increasingly strives to screen women for past-year IPV^65^ though uptake of IPV screening is variable.^66, 67^ Pre- and post-military SV and lifetime IPV are not routinely assessed within VHA. Recent legislation^68^ requires VHA to pilot services to address IPV *and* pre- and post-military SV (in addition to MST). Our findings reinforce the importance of these ongoing efforts and underscore the utility of expanding existing screening protocols and associated treatments. Additionally, the current findings respond to legislation calling for a better understanding of the prevalence of IPV and SV among veterans, particularly women and under-studied groups.^69^ Greater knowledge of these ubiquitous experiences can inform comprehensive SV and IPV detection and intervention approaches within VHA, the Department of Defense, and other healthcare systems, thereby facilitating increased disclosures of violence across the lifespan and enabling linkages to effective treatments.

Our findings also apply to general internal medicine settings, as veterans oftentimes prefer using non-VHA services for internal medicine.^70^ Findings can increase knowledge and awareness about the ubiquity of SV and IPV experiences among veterans among internists and facilitate trauma-informed SV and IPV inquiry, including queuing providers to consider whether certain veterans may be at increased risk (e.g., women and sexual minorities). SV and IPV screening is important for identifying patients at high risk for mental health difficulties.^4, 48^ PTSD is one of the most common impacts of SV and IPV,^71^ and it is associated with worse outcomes; yet nearly 30% of patients with PTSD go undetected in secondary clinics.^72^ Moreover, those who experience SV before or during military service are at increased risk of revictimization; thus, screening and referrals to therapeutic services can allow for intervention before additional traumas occur.^73, 74^
